# The N-terminal domain of the endoplasmic reticulum-resident chaperone Grp94 functions in aggregation prevention

**DOI:** 10.1016/j.jbc.2026.113358

**Published:** 2026-07-22

**Authors:** Ayodeji Adedeji, Erin Unruh, Alyssa Derr, Chioma Ndolo, Melina Balch, Kylie Paul, Abraham J. Morton, Andrea N. Kravats

**Affiliations:** 1 Department of Chemistry & Biochemistry, Miami University, Oxford, Ohio, USA; 2 Cell, Molecular, and Structural Biology Graduate Program, Miami University, Oxford, Ohio, USA

**Keywords:** aggregation prevention, BiP, chaperone, Grp94, peptide-binding

## Abstract

Glucose-regulated protein 94 (Grp94), an Hsp90 molecular chaperone localized in the endoplasmic reticulum, plays a central role in maintaining cellular proteostasis by facilitating protein folding and refolding in a nucleotide-dependent manner. Beyond its folding functions, Grp94 also prevents the aggregation of client proteins under stress conditions; however, the mechanistic basis of this aggregation-prevention activity is not fully understood. In this study, we investigated the requirements of Grp94’s prevention of aggregation activity and identified the functional domain responsible using *in vitro* light scattering and functional assays with two model client proteins, citrate synthase and luciferase. The results show that Grp94 suppresses the thermal aggregation of clients independently of ATP-binding, ATP-hydrolysis and calcium binding. Notably, the aggregation prevention activity for both clients is localized to the N-terminal domain of Grp94, where a peptide-binding deficient mutant (H146D) shows enhanced aggregation suppression at limiting chaperone concentrations. This enhanced prevention of aggregation activity is attributed to increased affinity for misfolded clients, which is likely due to decreased client dissociation rates. Consequently, client release by the Grp94 peptide-binding mutant is inhibited, thereby impairing downstream remodeling by the BiP chaperone system. While the N-terminal domain of Grp94 appears to be a general requirement in the prevention of aggregation, the involvement of Grp94’s pre-N domain is likely client specific. Collectively, these findings provide new insights into the aggregation-prevention function of Grp94 and implicate a previously identified peptide-binding site in aggregation prevention activity.

Molecular chaperones serve diverse roles in protein quality control, coordinating activities that range from protein folding and targeted degradation to protecting cells against protein aggregation and clearing these toxic aggregates (1, 2, 3). The failure in these processes has been implicated in numerous diseases, including neurodegenerative disorders such as Alzheimer's and Parkinson's diseases, as well as in metabolic conditions and numerous types of cancers (3, 4, 5). Hsp70 and Hsp90 chaperone systems function as central hubs in protein homeostasis, aiding in client protein recognition, folding, remodeling, and quality control (6, 7, 8).

Hsp90 is a highly conserved and abundant molecular chaperone that is central to protein homeostasis. Many client proteins, including kinases, transcription factors, and aggregation-prone proteins implicated in cancer and neurodegeneration, depend on Hsp90 to preserve their native conformation and functional activity (9, 10, 11, 12). It has been well established that Hsp90 actively folds, stabilizes, and reactivates non-native conformations of client proteins (13, 14). This function is coupled with Hsp90’s ATP binding and hydrolysis activity that results in conformational transitions within the chaperone (10, 15).

Beyond its ATP-driven folding cycle, Hsp90 has an ATP-independent “holdase” activity that involves binding to misfolded or non-native clients, thereby preventing their aggregation and maintaining them in a soluble, folding-competent state (6, 9, 16, 17). Hsp90s preferentially target intrinsically disordered regions across a large fraction of the proteome, helping to keep these proteins from misfolding, aggregating, or transitioning into aberrant condensates, such as stress granules and P-bodies (11, 12, 18). In Parkinson’s disease models, Hsp90 inhibits aggregation of a pathological mutant of α-synuclein. By tightly binding transient toxic oligomers in an ATP-independent manner, Hsp90 blocks fibril formation and converts the oligomers into nontoxic, soluble species, (9, 19, 20). A similar ATP-independent binding mechanism applies to Hsp90 and other amyloid-forming clients like Tau, where Hsp90 binds to aggregation-prone regions to inhibit mature fibril formation of Tau (21). Recent studies have shown that a small β-hairpin peptidomimetic modeled after the Tau-interacting region of Hsp90 can effectively inhibit the formation of Tau fibrils (22).

Glucose-regulated protein 94 (Grp94) is the endoplasmic reticulum (ER) paralog of the Hsp90 family of molecular chaperones (23, 24). Grp94 is constitutively expressed in multicellular organisms and is upregulated in response to ER stress, where it helps maintain ER proteostasis by assisting in protein folding, regulating ER calcium storage, and assisting in the removal of misfolded proteins through ER-associated degradation (24, 25). Unlike other ER chaperones such as BiP or calreticulin, Grp94 is a relatively selective chaperone that is essential for the folding, assembly, and maturation of a specific subset of secretory and membrane proteins, including integrins, Toll-like receptors, and insulin-like growth factors (24, 26, 27).

Grp94 assembles as a homodimer, with each monomer comprising an N-terminal domain (NTD), a middle domain (MD), and a C-terminal domain (CTD), with the NTD connected to the MD by a long acidic linker. While nucleotides bind within the NTD, residues located in both the NTD and MD coordinate nucleotide binding and hydrolysis (28). The MD and CTD also harbor residues that are hypothesized to be important for client interactions (29, 30). In addition to this canonical Hsp90 architecture, both mitochondrial and ER Hsp90 paralogs, TRAP1 and Grp94, respectively, contain an extended N-terminal pre-N domain, a feature that is much shorter or absent in cytosolic Hsp90s (30, 31, 32). This pre-N domain plays a critical role in regulating Grp94's conformational dynamics by slowing dimer closure and reducing ATP hydrolysis rates (32). Additionally, the pre-N domain has been suggested to be vital for both client binding and activation of Grp94 (30, 33).

Grp94’s function *in vivo* is tightly coordinated with its ATPase activity, as mutational analyses demonstrate that both ATP binding and hydrolysis are required for Grp94’s ability to support the active folding and maturation of key clients, such as IGF-II, and to maintain cell viability under stress conditions (34). Independent of its chaperone function, Grp94 also possesses peptide-binding activity, which is nucleotide independent and regulated by calcium binding (35, 36). Biochemical studies have mapped a peptide-binding site within Grp94’s NTD (37, 38, 39). However, these studies used isolated peptides in the absence of other ER factors, so the extent to which this site mediates binding of full-length clients in the cell remains unclear. In contrast, genetic and functional studies in cells show that this peptide-binding activity is not strictly required for Grp94’s essential chaperone function *in vivo* (34), suggesting that distinct domains and activities of Grp94 are specialized for different aspects of client handling (3, 27).

*In vivo* and in cell-based systems, Grp94 collaborates with BiP to support client refolding and maturation, yet studies of Toll-like receptors, integrins, and immunoglobulin heavy chains show that this collaboration is client-specific rather than universally required (40, 41, 42). More broadly, Hsp90 proteins, including Grp94, mediate the refolding and reactivation of client proteins, often acting downstream of Hsp70s to ensure efficient client maturation and recovery under stress conditions (6, 9, 10, 43). This functional partnership involves direct interactions between Hsp90 and Hsp70, with Hsp70 binding and stabilizing unfolded proteins before transfer to Hsp90 for final folding and activation (6, 44, 45).

Recent studies indicate that under severe stress promoting protein aggregation, Grp94 can act upstream of BiP by preventing aggregation of misfolded proteins, whereby Grp94 functions to transiently hold the client proteins in a soluble state. After the stress is removed and BiP is available, clients can be transferred to BiP for refolding and activation (46). This generalized Hsp90 activity in prevention of aggregation is ATP-independent and does not require the presence of Hsp70s (6, 9). In this study, we sought to define the role of Grp94 in aggregation prevention and to identify the underlying mechanisms. To this end, we utilized a well-established aggregation-prevention assay to assess which Grp94 domains are responsible for aggregation suppression using two model client proteins, citrate synthase (CS) and luciferase. Using fixed-angle and dynamic light scattering assays, we investigate how Grp94 suppresses client protein aggregation and thermal inactivation. By dissecting Grp94 into its constituent domains, these studies aim to define the regions responsible for aggregation prevention and to localize potential client interaction sites.

## Results

### Full-length Grp94 prevents the aggregation of chemically and thermally denatured citrate synthase in a concentration dependent manner

**Figure 1:**
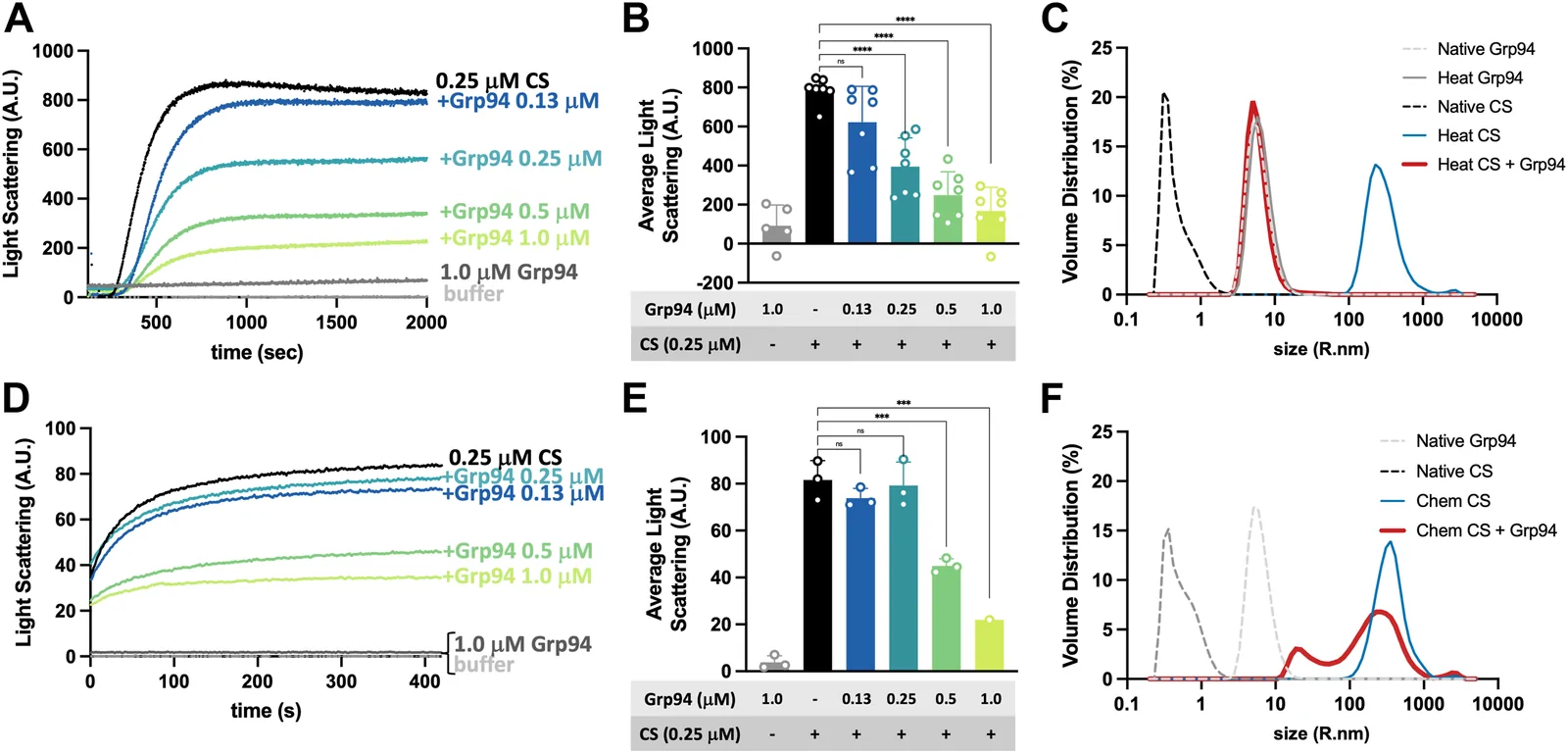
**Grp94 prevents aggregation of thermally and chemically denatured citrate synthase.***A*, Citrate Synthase (CS) (0.25 μM) was incubated at 45 °C for 30 min alone or in the presence of Grp94 (0.125 μM, 0.25 μM, 0.5 μM, and 1 μM) and 90 °C light scattering was recorded as a function of time. A representative time trace from five to seven independent experiments is shown. *B*, the plateau values for each curve in (*A*) were averaged from 1500 to 1800 s and are shown in bar graphs as mean ± SD, with individual data points representing each independent experiment. Statistical significance was assessed by an ordinary one-way ANOVA (F(4,30) = 26.22, *p* < 0.0001), followed by Dunnett’s multiple comparisons test comparing each condition to the CS alone control group. Exact adjusted *p-*values for individual comparisons are indicated by asterisks: ∗∗∗∗*p* < 0.0001; ns, not significant (*p* = 0.0905). *C*, dynamic light scattering of thermally denatured CS (0.25 μM) and Grp94 (1 μM). Native CS and Grp94 are included as controls. Data are presented as the mean of independent experiments (n = 19 for native or heated CS, n = 10 for native or heated Grp94, and n = 9 for CS + Grp94). *D*, chemically denatured CS (0.25 μM) was diluted into refolding buffer alone or in the presence of Grp94 (0.125 μM, 0.25 μM, 0.5 μM, and 1 μM) at room temperature and 90 °C light scattering was recorded as a function of time. A representative time trace from three independent experiments is shown. *E*, the plateau values for each curve in (*D*) were averaged from 240 to 360 s and are shown in bar graphs as mean ± SD, with individual data points representing independent experiments. Statistical significance was assessed by an ordinary one-way ANOVA (F(4,8) = 24.25, *p* = 0.0002), followed by Dunnett’s multiple comparisons test comparing each condition to the CS alone control group. Exact adjusted *p*-values for individual comparisons are indicated by asterisks: ∗∗∗*p* < 0.001 (explicitly, *p* = 0.0007 and *p* = 0.0003 for 0.5 μM and 1 μM Grp94, respectively); not significant (*p* = 0.5227 and *p* = 0.9826, respectively). *F*, dynamic light scattering of chemically denatured citrate synthase (0.25 μM) and Grp94 (1 μM). Data are presented as the mean of independent experiments (n = 12 for native or heated CS, n = 9 for native or heated Grp94, and n = 9 for Grp94+CS). CS, citrate synthase.

The prevention of aggregation ability of Grp94 was monitored for chemically or thermally denatured CS using fixed-angle light scattering (Experimental Procedures). In these experiments, CS was added to the refolding buffer, with or without chaperones, and fixed-angle light scattering was monitored. Increases in light scattering signal indicate the presence of aggregated CS, while decreases in light scattering signal when Grp94 is included in reaction mixtures indicate the Grp94-mediated prevention of CS aggregation. In agreement with previous observations, thermally denatured CS in the absence of chaperones is observed to aggregate (47, 48), while Grp94 alone does not significantly aggregate under these conditions (Fig. 1, *A* and *B*). When CS is added into the refolding buffer containing increasing amounts of Grp94 present, the prevention of CS aggregation is observed (Fig. 1, *A* and *B*). Similarly, Grp94 prevents the aggregation of chemically denatured CS in a concentration dependent manner (Fig. 1, *D* and *E*).

To better understand the effect of Grp94 on aggregation, dynamic light scattering (DLS) was used to characterize the size of aggregates. In control experiments, native CS has an approximate size of 1 nm, while native Grp94 has an approximate size of 6 nm (Fig. 1, *C* and *F*). Thermally denatured CS forms large aggregates with an approximate hydrodynamic radius size of 300 to 400 nm. The inclusion of Grp94 wild type (WT) in reaction mixtures reduces the size of thermally denatured CS aggregates to the size of Grp94 alone (Fig. 1*C*), suggesting denatured CS is held in complex with Grp94. A similar trend is observed for chemically denatured CS, with the presence of Grp94 reducing aggregate size to approximately 17 nm (Fig. 1*F*). Together, similar patterns for aggregation prevention of thermally and chemically denatured CS by Grp94 were observed (Fig. 1). However, Grp94 was not able to fully resolve the formation of chemically-induced aggregates when compared to heat-induced aggregates. This difference in aggregation prevention likely results from aggregation kinetics, as thermal denaturation of CS results in the slow formation of dimeric aggregates, while chemical denaturation leads to rapidly aggregating monomeric species with a larger aggregate size (48). Given that Grp94’s ability to prevent aggregation appears to be limited by the rate of chemical aggregation, we focused exclusively on the thermal denaturation model in the subsequent experiments.

We questioned whether Grp94, during the prevention of aggregation, was holding the client in a reactivatable intermediate configuration or whether the client was misfolded and kinetically trapped. To test this, we implemented a citrate synthase activity assay that measures the enzyme’s capacity to catalyze the reaction of oxaloacetate and acetyl-CoA, producing citrate and free CoA-SH. The production of CoA-SH is monitored spectrophotometrically through the addition of 5,5′-Dithiobis-(2-nitrobenzoic acid) (DTNB), which reacts with the free thiol of CoA-SH. During the prevention of aggregation assay, aliquots of the reaction mixture were removed at various timepoints and tested for CS activity. The presence of Grp94 during the thermal denaturation promoted sustained CS activity, suggesting that Grp94 not only prevents aggregation but also holds the client in a moderately reactivatable conformation (Fig. S1).

### Aggregation prevention by Grp94 does not require calcium binding nor ATP binding or hydrolysis

**Figure 2:**
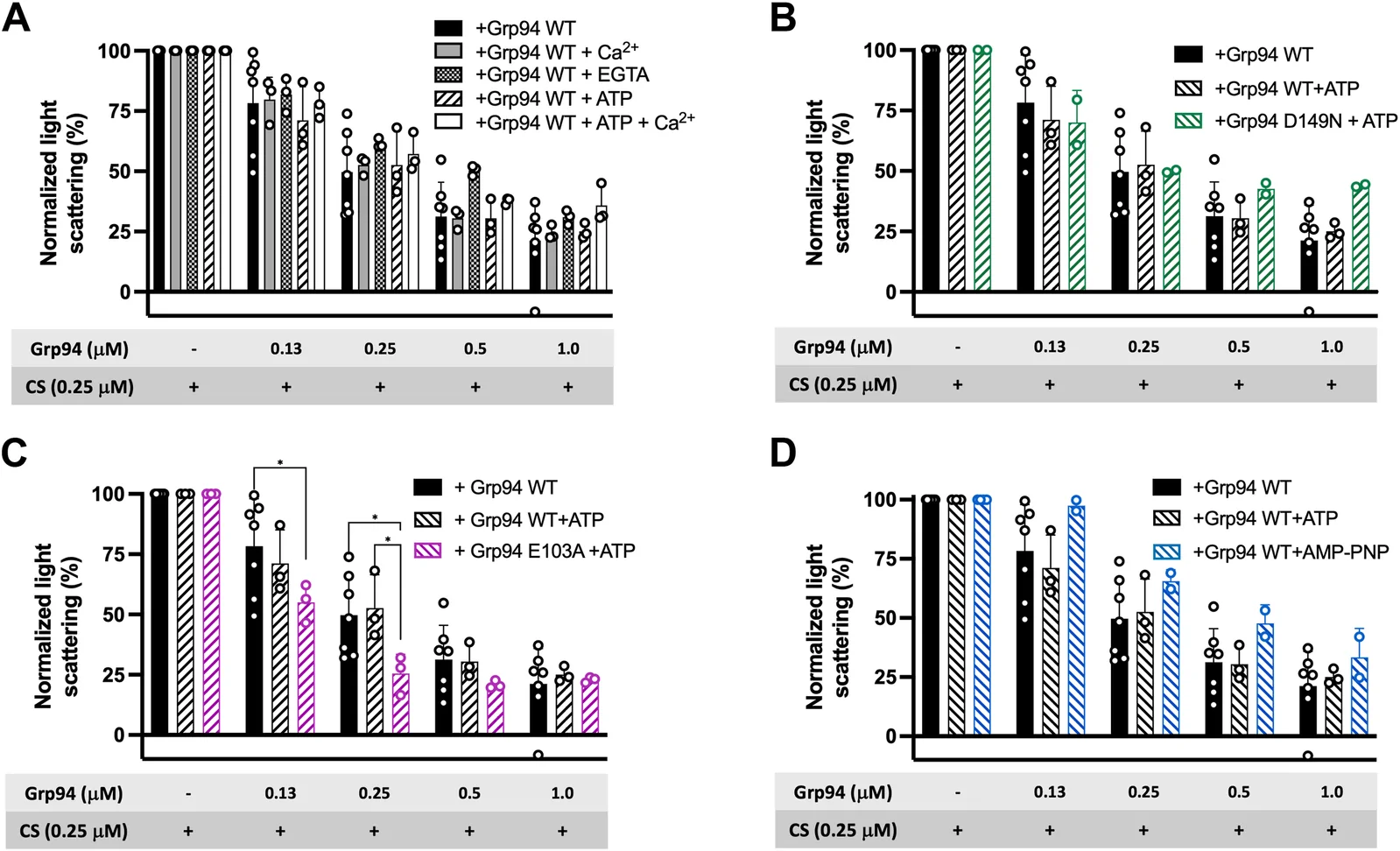
**Aggregation prevention by Grp94 is independent of calcium binding, nucleotide binding and hydrolysis, and Grp94’s conformation.** Fixed-angle light scattering assays were carried out as described in Methods. Citrate synthase (CS) (0.25 μM) was incubated at 45 °C for 30 min alone or in the presence of WT or mutant Grp94 (0.125 μM, 0.25 μM, 0.5 μM, 1 μM). The plateau values for each time trace were averaged and are shown in bar graphs as mean ± SD (n = 7 for Grp94 WT without additives and n = 3 for Grp94 mutants or WT with additives), with individual data points representing independent experiments. *A*, effect of calcium, ethylene glycol tetracetic acid, and ATP on the prevention of citrate synthase aggregation by WT Grp94. Reactions were supplemented with 1 mM Ca^2+^, 1 mM ethylene glycol tetracetic acid, 50 μM ATP, or both ATP and calcium, as indicated. *B*, prevention of citrate synthase aggregation by Grp94 D149N with ATP compared to WT Grp94 with or without ATP. *C*, prevention of citrate synthase aggregation by E103A Grp94 with ATP compared to WT Grp94 with or without ATP. *D*, prevention of citrate synthase aggregation by WT Grp94 incubated with 10 mM AMP-PNP compared to WT Grp94 with or without ATP. For (*A* and *D*), statistical variance was analyzed using ordinary two-way ANOVAs. For (*A*) the analysis revealed a significant effect for the Grp94 concentration (F (4,70) = 129.8, *p* < 0.0001 but no significant effect for the treatment conditions (F(4, 70) = 2.37, *p* = 0.0604) or interaction between Grp94 concentration and condition (F(16, 70) = 0.49, *p* = 0.9457). Pairwise comparisons within each Grp94 concentration were performed using Tukey’s multiple comparisons test; all comparisons within each concentration group showed no statistically significant differences (*p* > 0.0885 across all pairs). For (*B*), the analysis revealed a significant effect for the Grp94 concentration (F(4,45) = 45.83, *p* < 0.0001) but no significant effect for the Grp94 variant or treatment conditions (F(2,45) = 0.6931, *p* = 0.5053) or interaction between Grp94 concentration and condition (F(8,45) = 0.7309, *p* = 0.6636). Pairwise comparisons within each Grp94 concentration were performed using Tukey’s multiple comparisons test; all comparisons within each concentration group showed no statistically significant differences (*p* > 0.0853 across all pairs). For (*C*), the analysis revealed a significant effect for the Grp94 concentration (F(4,50) = 73.92, *p* < 0.0001) and the Grp94 variant or treatment conditions (F(2,50) = 4.680, *p* = 0.0137) but no significant effect for the interaction between Grp94 concentration and condition (F(8,50) = 1.169, *p* = 0.3361). Pairwise comparisons within each Grp94 concentration were performed using Tukey’s multiple comparisons test; significant individual pairs are indicated by *asterisks*: ∗*p* < 0.05 (explicitly, *p* = 0.0240 at 0.13 μM; *p* = 0.0183 and *p* = 0.0266 at 0.25 μM, respectively). All other comparisons within each concentration group were non-significant (*p* > 0.25). For (*D*), the analysis revealed a significant effect for the Grp94 concentration (F(4,47) = 60.64, *p* < 0.0001) and the treatment condition (F(2,47) = 4.567, *p* = 0.0154) but no significant effect for the interaction between Grp94 concentration and condition (F(8,47) = 0.5828, *p* = 0.7868). Pairwise comparisons within each Grp94 concentration were performed using Tukey’s multiple comparisons test; all comparisons within each concentration group were nonsignificant (*p* > 0.0721). CS, citrate synthase.

Grp94 assists in calcium storage and signaling within the ER by directly binding calcium (49). Additionally, as part of its client remodeling mechanism, Grp94 binds and hydrolyzes ATP (34). Thus, we sought to determine the effects of calcium and ATP binding on CS aggregation prevention by Grp94. We began by supplementing the reaction mixtures with Ca^2+^. At all Grp94 concentrations tested, there was no significant effect observed in Grp94’s prevention of aggregation activity (Figs. 2*A* and S2, *A*, *B*). However, recognizing that the experiments with no added calcium likely contain residual calcium, we introduced the chelating agent ethyleneglycol-*bis*(β-aminoethyl)-N,N,Nʹ,Nʹ-tetraacetic acid to reduce calcium. Under these conditions, Grp94 appeared to be less effective at preventing aggregation at higher concentrations of chaperone, though these results were not statistically significant (Figs. 2*A* and S2, *C*, *D*). Including ATP in the reactions showed little effect on prevention of aggregation (Figs. 2*A* and S2, *E*, *F*). Similarly, supplementing with ATP and calcium showed slight decreases in prevention of aggregation, though the results are not statistically significant (Figs. 2*A* and S2, *G*, *H*).

To confirm that nucleotide binding and hydrolysis are not necessary for the prevention of aggregation activity, we tested the Grp94 ATP-binding defective mutant, D149N, and the ATP hydrolysis defective mutant, E103A (Fig. S3*A*). In control experiments, these mutants were shown to fold similarly to Grp94 WT (Fig. S3, *B* and *C*). There were no significant differences observed in aggregation prevention for the ATP-binding defective mutant compared to Grp94 WT (Figs. 2*B* and S4, *A*, *B*). However, at sub-stoichiometric or stoichiometric chaperone concentrations, the ATP-binding defective mutant showed slightly enhanced ability to prevent aggregation (Figs. 2*C* and S4, *C*, *D*). Together, these results suggest that prevention of aggregation by Grp94 is nucleotide and calcium independent, since aggregation prevention activity is preserved under the conditions tested.

Since nucleotide did not play an important role in Grp94’s ability to prevent aggregation, we questioned whether Grp94’s global conformation was important for the prevention of aggregation. In contrast to other Hsp90s, the conformations adopted by Grp94 are shown to be relatively insensitive to nucleotide, with Grp94 populating flexible open conformations even in the presence of ATP (40, 50, 51). Incubation of full-length Grp94 with AMP-PNP, a non-hydrolysable ATP analog, results in an increased population of the catalytically active conformation (40), characterized by the closing of the protomers around the hypothesized client binding pocket and dimerization of the NTDs (30). We preincubated Grp94 with AMP-PNP and tested for the prevention of aggregation. The results suggest that the closed Grp94 conformation is less effective at preventing aggregation, though these results are not statistically significant (Figs. 2*D* and S4, *E*, *F*). These findings suggest that all solution forms of Grp94 are effective at preventing client aggregation.

### The N-terminal domain of Grp94 interacts with client proteins to prevent aggregation

We were interested in identifying which region of Grp94 was responsible for the prevention of aggregation activity, since previous studies yielded conflicting outcomes. One study reported that the CTD was important for preventing aggregation of the catalytic subunit of protein kinase CK2, but not for malate dehydrogenase (52). A crystal structure of the catalytically active conformation of Grp94 captured an unstructured polypeptide client-mimetic interacting in an internal channel formed by residues in the MD and CTD of Grp94 (30). Alternatively, a recent study with myocilin showed that regions in Grp94’s MD protected against aggregation, while residues in the pre-N and NTD promoted aggregation (33). Therefore, we probed which Grp94 domain is responsible for preventing aggregation by engineering and purifying isolated domains, including the NTD (23–287), MD (337–594), and CTD (581–803), and testing for prevention of aggregation activity.

**Figure 3:**
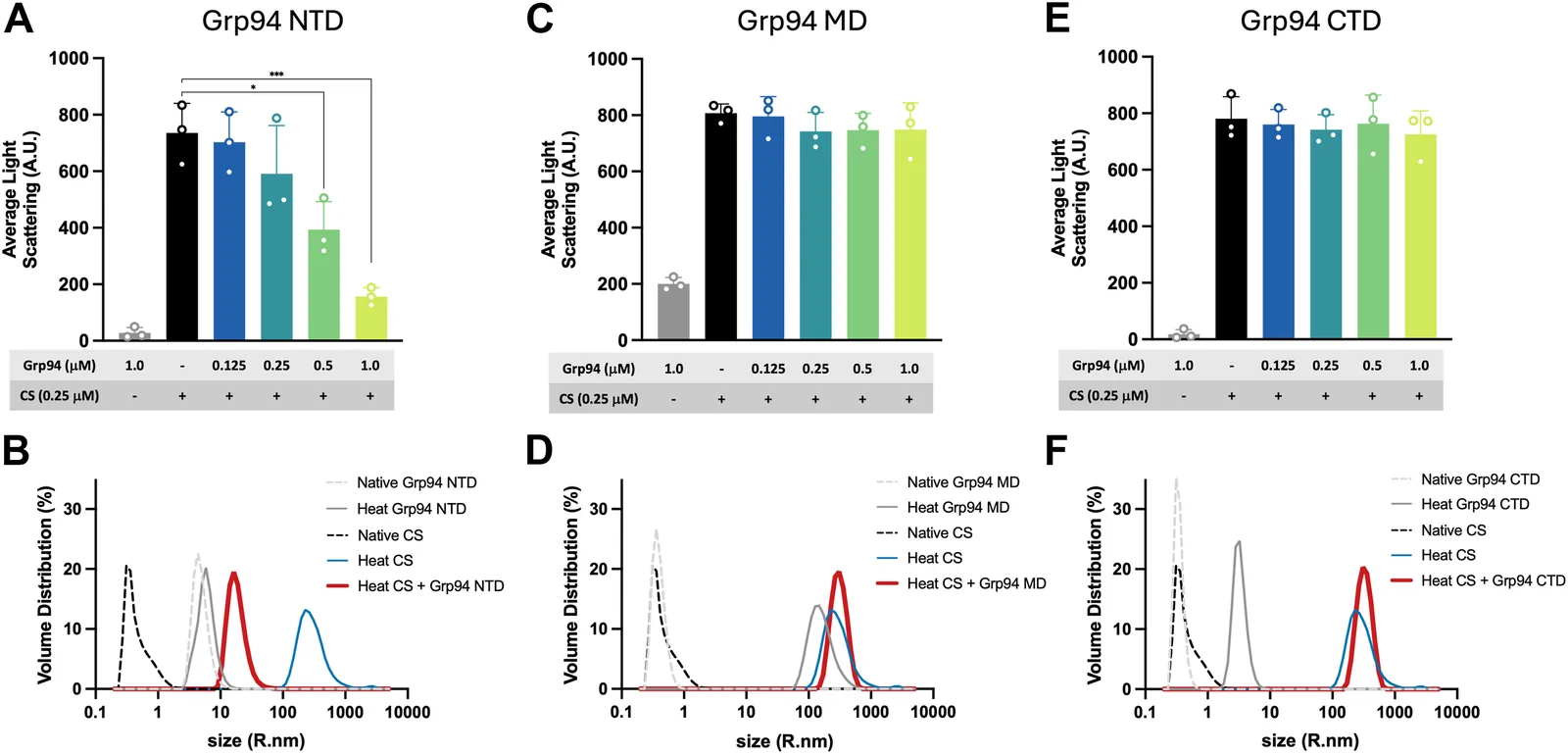
**The NTD of Grp94 is responsible for citrate synthase aggregation prevention.***A*, *C*, and *E*, fixed-angle light scattering assays were carried out as described in Methods. CS (0.25 μM) was incubated at 45 °C for 30 min alone or in the presence of Grp94 domains (0.125 μM, 0.25 μM, 0.5 μM, 1 μM); (*A*) Grp94 NTD, (*C*) Grp94 MD, (*E*) Grp94 CTD. The plateau values for each time trace were averaged and are shown in bar graphs as mean ± SD (n = 3 independent experiments), with individual data points representing independent experiments. *B*, *D*, and *F*, dynamic light scattering of 0.25 μm of heat denatured CS and 1 μM of Grp94 domains; (*B*) Grp94 NTD, (*D*) Grp94 MD, (*F*) Grp94 CTD. Data are presented as the mean of independent experiments (n = 19 for native and heated CS; n = 3 for all other conditions). For *A*, *C*, and *E*, statistical variance was analyzed using ordinary one-way ANOVAs followed by Dunnett’s multiple comparisons test comparing each condition to the CS alone control group. For (*A*), (F(4,10) = 13.99, *p* = 0.0004), Exact adjusted *p*-values for individual comparisons are indicated by *asterisks*: ∗∗∗*p* = 0.0003, ∗*p* = 0.0124, not significant (*p* = 0.9886 and 0.3708, respectively). For (*C*), (F(4,10) = 0.6117, *p* = 0.6638), all comparisons against the control showed no statistically significant differences (*p* > 0.6110). For (*E*), (F(4,10) = 0.2378, *p* = 0.9106), all comparisons against the control showed no statistically significant differences (*p* > 0.7796). CS, citrate synthase; CTD, C-terminal domain; NTD, N-terminal domain.

When the individual domains of Grp94 were purified, we observed some discrepancies in the solution-state properties of the individual domains, namely the NTD. Despite lacking the C-terminal dimerization domain, the NTDs undergo dynamic self-association to form a predominant population of oligomers in solution (Fig. S5). In control experiments, the NTD displayed little aggregation propensity on its own or when heated, ruling out the formation of dense insoluble aggregates (Figs. 3*A* and S6*A*). Conversely, the NTD exhibited a larger than expected hydrodynamic radius with peak sizes around 5 to 6 nm in dynamic light scattering experiments (Fig. 3*B*). We generated monomer and dimer models of Grp94’s NTD using AlphaFold and predicted the hydrodynamic radius using HullRad. Because these models partially compacted the pre-N domains within the NTDs, this yielded smaller theoretical values of hydrodynamic radius (2.9–4.3 nm) compared to the experimentally observed size of 5 to 6 nm. This likely reflects a highly dynamic and expanded configuration of the pre-N domains in solution. As the unanchored pre-N domain tumbles, it may sweep a large volume while sampling dimeric or oligomeric states, which is supported by experimental observations for other Hsp90s. While cytosolic Hsp90s typically purify as monomers, the inclusion of the charged linker drives it to assemble as dimers (53). Similarly, organelle-localized Hsp90s contain unusually long pre-N domains with charged features that may serve a similar function as the charged linker in the dimerization of cytosolic Hsp90. Indeed, mitochondrial Hsp90s lacking the C-terminal dimerization domain form intertwined dimers (54), while isolated NTDs containing the pre-N domains dimerize, with the pre-N domains demonstrating conformational plasticity by taking on multiple solution configurations (55). We propose that Grp94’s NTDs predominantly form oligomeric species in solution due to the presence of the highly flexible pre-N domain that is capable of driving conformational expansion and self-assembly. In contrast, the MD and CTDs migrate as monomers (Fig. S5). The MD in its native form is stable but moderately aggregates when heated, as characterized by fixed angle and dynamic light scattering (Figs. 3, *C*, *D* and S6*B*). In contrast, Grp94 CTDs remain soluble in their native form and form soluble assemblies that are characteristic of oligomeric species (Figs. 3, *E*, *F* and S6*C*), consistent with previous reports that Grp94’s CTD can self-associate (35, 52).

When the NTD of Grp94 was tested in the prevention of aggregation assay, it demonstrated concentration-dependent prevention of aggregation similar to Grp94 full-length (Figs. 3*A* and S6*A*). This similar potency was observed when comparing equal concentrations of full-length Grp94 dimer and NTD monomer. While the addition of the NTD suppressed large-scale aggregation characterized by large hydrodynamic radius, DLS analysis revealed that the resulting NTD-CS complex maintained a larger hydrodynamic radius compared to native CS or Grp94 NTDs alone (Fig. 3*B*). This expanded radius was not observed when the full-length chaperone was present (Fig. 1*C*). In contrast to the observed prevention of aggregation by the NTD, isolated Grp94 MD and CTDs have no significant effect on preventing CS aggregation at all concentrations tested (Figs. 3, *C*, *E* and S6, *B*, *C*). DLS analysis showed that when CS was incubated with MD or CTD, the hydrodynamic radius remained identical to CS alone in the presence of the MD or CTD, though the peak height increased while the peak at the respective isolated domain size disappeared. These results suggest that the domains are recruited into the client aggregates (Fig. 3, *D* and *F*).

**Figure 4:**
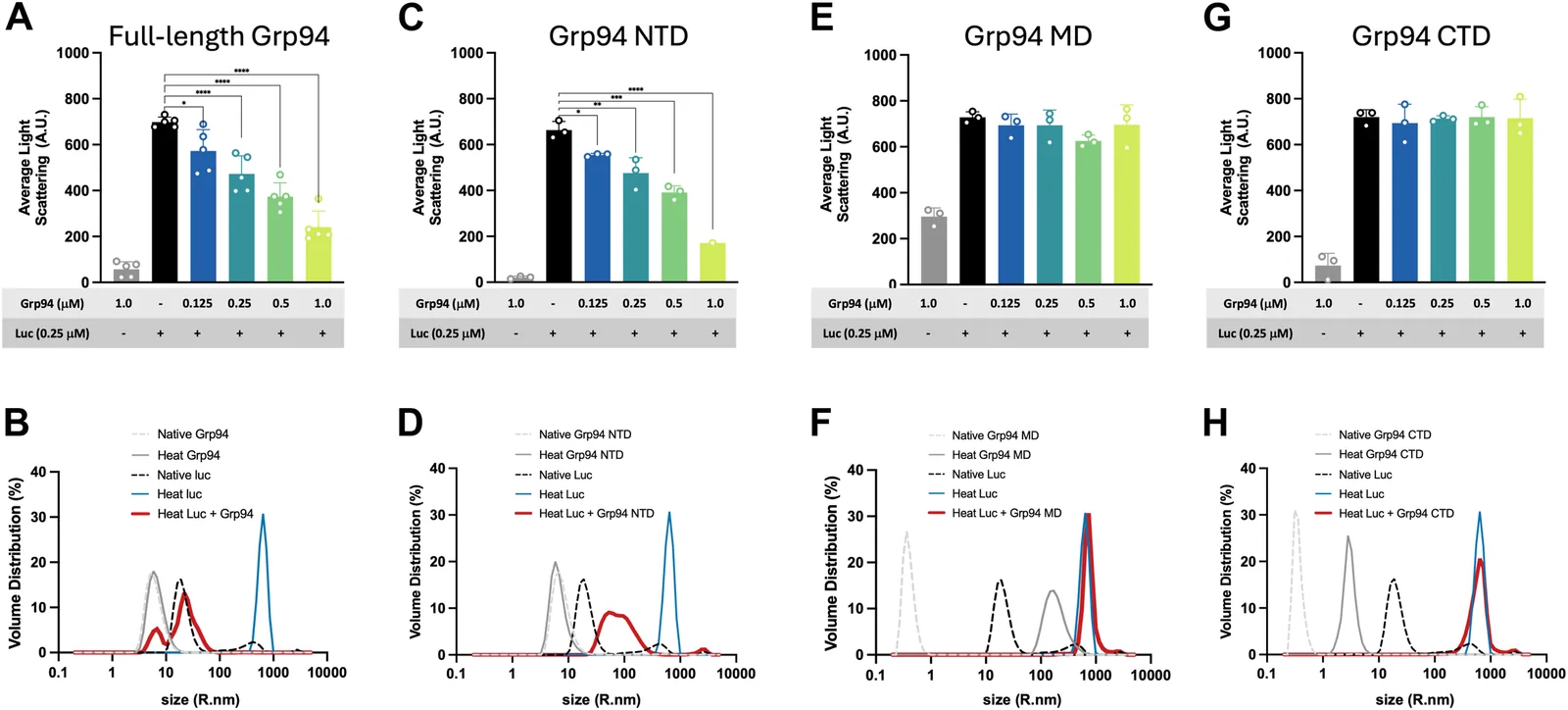
**The NTD of Grp94 is responsible for luc aggregation prevention.***A*, *C*, *E*, *G*, fixed-angle light scattering assays were carried out as described in Methods. Luc (0.25 μM) was incubated at 45 °C for 30 min alone or in the presence of Grp94 domains (0.125 μM, 0.25 μM, 0.5 μM, 1 μM); (*A*) full-length Grp94, (*C*) Grp94 NTD, (*E*) Grp94 MD, (*G*) Grp94 CTD. The plateau values for each time trace were averaged and are shown in bar graphs as mean ± SD (n = 5 for Grp94 full-length; n = 3 for the isolated domains), with individual data points representing independent experiments. (*B, D, F, H*) Dynamic light scattering of heat denatured luc (0.25 μM) and Grp94 domains (1 μM); (*B*) Grp94 full-length, (*D*) Grp94 NTD, (*F*) Grp94 MD, (*H*) Grp94 CTD. Data are presented as mean of independent experiments (n = 9 for native or heated luc; n = 3 for all other conditions). For *A*, *C*, *E* and *G*, statistical variance was analyzed using ordinary one-way ANOVAs followed by Dunnett’s multiple comparisons test comparing each condition to the CS alone control group. For (*A*), (F(6,28) = 95.03, *p* < 0.0001), Exact adjusted *p*-values for individual comparisons are indicated by asterisks: ∗*p* = 0.0127, ∗∗∗∗*p* < 0.0001. For (*C*), (F(4,8) = 34.52, *p* < 0.0001), ∗*p* = 0.0386, ∗∗*p* = 0.0018, ∗∗∗*p* = 0.0001, ∗∗∗∗*p* < 0.0001. For (*E*), (F(4,10) = 1.347, *p* = 0.3188), all comparisons against the control showed no statistically significant differences (*p* > 0.1408). For (*G*), (F(4,10) = 0.1061, *p* = 0.9777), all comparisons against the control showed no statistically significant differences (*p* > 0.9459). CTD, C-terminal domain; luc, luciferase; NTD, N-terminal domain.

To determine whether the prevention of aggregation by the NTD of Grp94 is a client-specific mechanism or more widely conserved, we carried out aggregation prevention assays with a different model client protein, luciferase. Luciferase readily aggregates under high temperatures, and aggregation prevention by Hsp90 has previously been demonstrated (56, 57). Grp94 has also been shown to prevent thermal aggregation of luciferase in an ATP-independent manner with downstream refolding by the BiP chaperone system (46). In line with previous observations, luciferase aggregates when thermally denatured and full-length Grp94 reduces luciferase aggregation (Figs. 4, *A* and *B* and S7*A*). Including the isolated Grp94 domains in the experiments revealed the NTD is responsible for aggregation prevention (Figs. 4, *C*, *D* and S7*B*), while luciferase aggregation is unaffected by the presence of Grp94 MD or CTDs (Figs. 4, *E*–*H* and S7, *C*, *D*). Similar to the observations with CS, the prevention of aggregation by the NTD’s does not completely revert the size of the luciferase aggregates to that of the size of Grp94 NTD as observed for the full-length chaperone. Instead, Grp94 NTD-luciferase complexes are formed that are larger than the Grp94 NTD alone but smaller than aggregated luciferase (Fig. 4, *B* and *D*). Consistent with the trend reported in CS aggregation prevention, the NTD forms higher-order oligomers, and the MD aggregates in the absence of CS during heat denaturation. Together, these results agree with the findings that Grp94’s NTD prevents aggregation and highlight the role of Grp94’s NTD in client binding for the prevention of aggregation activity.

**Figure 5:**
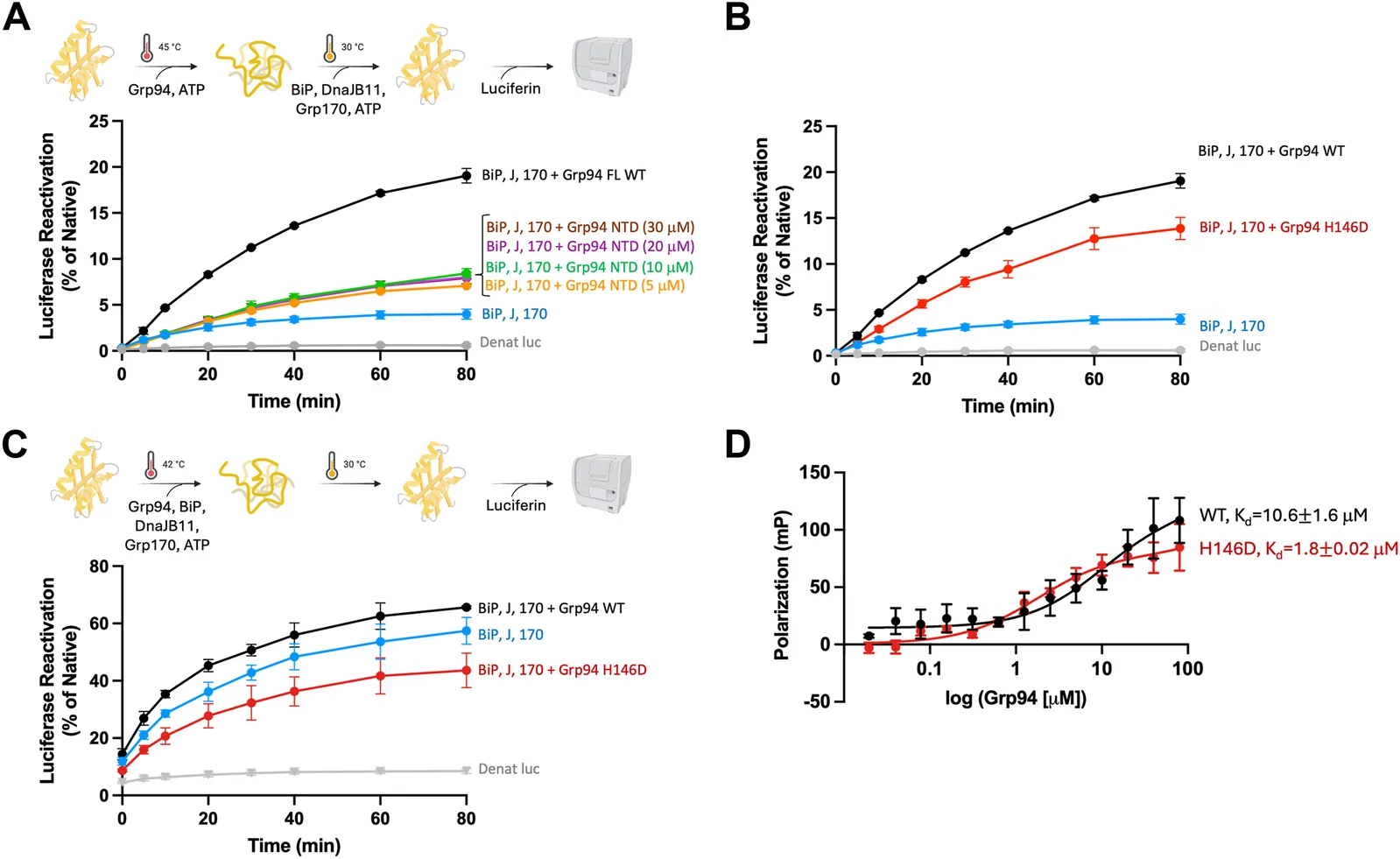
**Grp94 NTD or H146D prevents luciferasereactivation with BiP and cochaperones.***A*, Luciferase (Luc) (160 nM) was heat denatured in the presence or absence of 5uM WT Grp94 or Grp94 NTD (ranging from 5 to 30 μM, monomer equivalents) with ATP. Reactions were diluted 10-fold into buffer containing the BiP, DnaJB11 (*J*), and Grp170 (170), and luc reactivation was measured at the designated time points. *B*, the experiment from (*A*) was performed with full-length Grp94 WT and H146D. *C*, luc (25 nM) was heat denatured at 42 °C for 10 min in the presence of BiP, DnaJB11, Grp170, and Grp94 WT or H146D in buffer containing ATP. Reactions were diluted into buffer containing an ATP regenerating system to a final luc concentration of 20 nM and incubated at 30 °C, and activity was measured over time. Data shown represent the mean ± SD of four independent experiments (*A* and *B*) and three independent experiments (*C*). *D*, fluorescence polarization binding of FITC-labeled Δ131Δ K16C (50 nM) to Grp94 WT and H146D. Serial dilutions of Grp94 (0–80 μM) were incubated with Δ131Δ K16C for 30 min at 37 °C, and steady-state polarization was fit to a one-site specific binding model. Data shown represent the average of at two experiments for Grp94WT and three experiments for Grp94 H146D. The calculated binding affinity ± SD are indicated.

These results have demonstrated that the isolated NTD is sufficient to bind the client and maintain its solubility, thereby preventing aggregation; however, this observation only addresses the chaperone’s holding function. The functional refolding of clients requires collaboration with the BiP system. To test whether Grp94’s NTD was sufficient for remodeling, we utilized a luciferase remodeling assay that begins with the prevention of aggregation by Grp94 (46). In this assay, luciferase was denatured in the presence of either full-length Grp94 or Grp94 NTD and diluted into buffer containing BiP and BiP cochaperones for downstream refolding (Experimental Procedures). Since equivalent concentrations of full-length Grp94 dimer and Grp94 NTD monomer have similar effectiveness in suppressing aggregation (Figure 1, Figure 3, *A–C*, 3, *A–C*, S6, *A*, 4, *A*–*D* and S7 *A* and *B*), we evaluated these concentrations in the functional luciferase remodeling assay. This experimental design operates on the model that one Grp94 NTD is sufficient to interact with the client, even within the full-length dimer. Under these conditions, reactions containing the Grp94 NTD are deficient in luciferase remodeling, as a ∼2.5-fold reduction in reactivation is observed compared to reactions containing full-length Grp94 (Fig. 5*A*). To ensure that this observation was not a concentration-dependent artifact, we titrated the concentration of Grp94 NTD to attempt to rescue remodeling activity. The NTD concentration was extended to a 6-fold excess compared to full-length Grp94, yet luciferase remodeling activity remained defective under all the NTD concentrations tested (Fig. 5*A*). Together these results suggest that the NTD alone is sufficient for preventing aggregation but insufficient for active client remodeling.

### The peptide-binding region of Grp94 is involved in aggregation prevention

We were intrigued by the NTD’s role in preventing aggregation, especially since Grp94’s NTD includes a peptide-binding region known for interacting with immunogenic peptides (38, 58). Previous research has localized Grp94’s peptide-binding site to a region involving residue H146 (38, 59, 60). Mutation of this histidine to aspartate was shown to abolish direct physical interactions with two peptides, VSV8 and Pep A (38). Interestingly, peptide binding does not require ATP (35), though; peptide binding was reported to require Ca^2+^ binding (36). Given the interactions of CS with the NTD of Grp94 and the ATP and calcium independence of aggregation prevention, we questioned whether interactions with clients occur through Grp94’s peptide-binding region.

**Figure 6:**
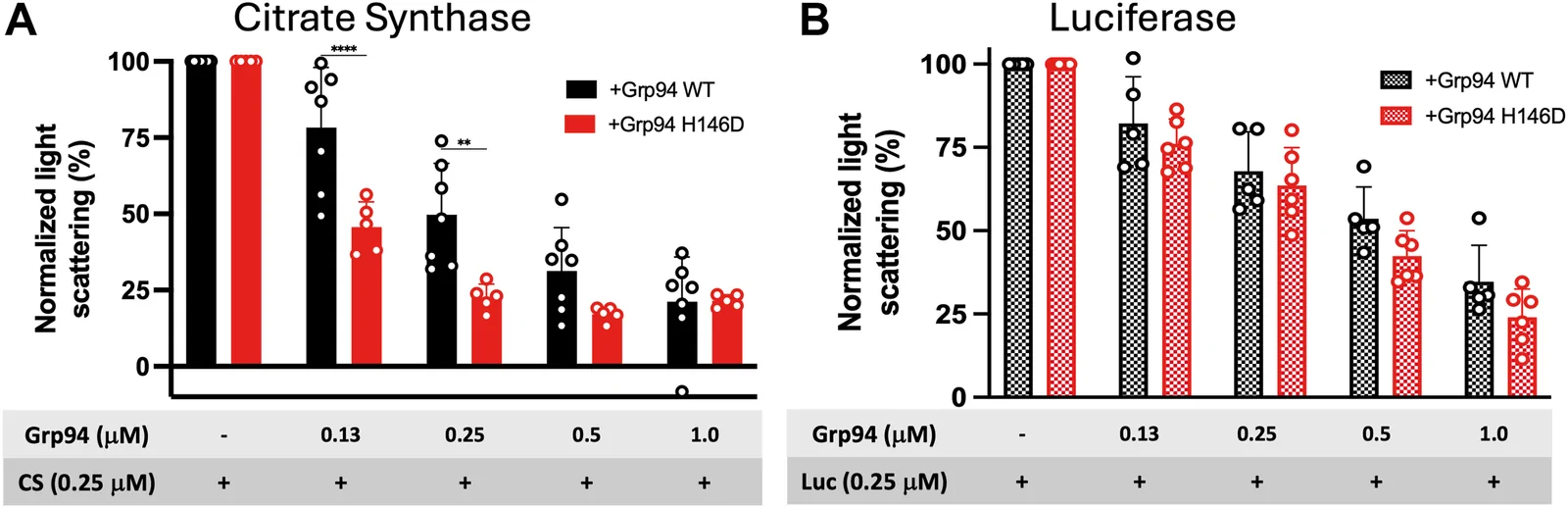
**The peptide-binding mutant of Grp94 exhibits enhanced aggregation prevention at limiting chaperone concentrations.** Fixed-angle light scattering assays were carried out as described in Methods. *A*, CS or (*B*) luc (0.25 μM) was incubated at 45 °C for 30 min in the absence or presence of WT Grp94 or Grp94 H146D (0.125 μM, 0.25 μM, 0.5 μM, or 1 μM). The plateau values for each time trace were averaged and are shown in bar graphs as mean ± SD, with individual data points representing independent experiments. In (*A*), n = 7 for Grp94 WT and n = 5 for Grp94 H146D; in (*B*), n = 5 for Grp94 WT and n = 6 for Grp94 H146D. For (*A*) and (*B*), statistical variance was analyzed using ordinary two-way ANOVAs. For (A) the analysis revealed significant effects for the Grp94 concentration (F (4,50) = 90.90, *p* < 0.0001), Grp94 variant (F(1, 50) = 22.86, *p* < 0.0001) and interaction between concentration and variant (F(4, 50) = 4.822, *p* = 0.0023). Pairwise comparisons between Grp94 WT and H146D at each chaperone concentration were performed using Tukey’s multiple comparisons test; significant individual pairs are indicated by asterisks: ∗∗∗∗*p* < 0.0001, ∗∗*p* = 0.0013. All other comparisons within each concentration group were non-significant (*p* > 0.2045). For (*B*), the analysis revealed a significant effect for the Grp94 concentration (F(4,45) = 97.15 *p* < 0.0001) and the Grp94 variant (F(1,45) = 6.798, *p* = 0.0123) but no significant effect for interaction between concentration and variant (F(4,45) = 0.7159, *p* = 0.5855). Pairwise comparisons within each Grp94 concentration group were performed using Tukey’s multiple comparisons test; all comparisons within each concentration showed no statistically significant differences (*p* > 0.2195 across all pairs). CS, citrate synthase; Luc, luciferase.

We generated the peptide-binding defective mutant, Grp94 H146D, and show that it folds similarly to Grp94 WT (Fig. S3, *A*–*C*). In aggregation prevention assays, Grp94 H146D is remarkably better at preventing CS aggregation compared to Grp94 WT, decreasing aggregation at low chaperone concentrations before the Grp94 concentration becomes saturating (Figs. 6*A* and S8*A*). DLS confirms the lack of formation of the larger CS aggregates when Grp94 H146D is present during denaturation, resulting in protection against aggregation through the formation of Grp94-CS complexes (Fig. S8*B*). Compared to Grp94 WT, Grp94 H146D also appears to be slightly more effective at preventing aggregation of heat-denatured luciferase, though these results are not statistically significant (Figs. 6*B* and S8, *C*, *D*).

### Grp94 H146D has a higher client binding affinity compared to Grp94 WT

We next questioned whether the improved prevention of aggregation by Grp94 H146D correlates with enhanced refolding in the presence of the BiP system. We utilized the same luciferase remodeling assay that was incorporated to test Grp94’s NTD (Fig. 5*A*). In reactions containing Grp94 H146D, luc reactivation is reduced 1.5-fold compared to those containing Grp94 WT (Fig. 5*B*).

In our previous work, we also observed the collaboration of Grp94 with the BiP chaperone system in luc refolding (40). The requirements for Grp94 are different from the previous assay in that ATP binding, ATP hydrolysis, and direct interactions with BiP are all required. In this assay, BiP, BiP cochaperones, Grp94, and ATP were all present during the denaturation step (Experimental Procedures). Similar to our previous observations, the presence of Grp94 WT enhanced refolding with the BiP system by ∼10% (40). However, the presence of Grp94 H146D not only failed to stimulate refolding with the BiP system but also resulted in hindered refolding compared to the BiP system alone (Fig. 5*C*). The effect of this mutation is very similar to that of Grp94 D149N in refolding, as Grp94 D149N exhibits a stronger client binding affinity that resulted in less reactivation (40).

These findings were surprising, because we anticipated that more effective prevention of aggregation would result in improved client reactivation. Thus, we hypothesized that Grp94 H146D interacted more strongly with clients compared to Grp94 WT, resulting in less luciferase being released by Grp94 for reactivation. To further examine differences in client binding, we analyzed the interaction of Grp94 WT and H146D with the model client Δ131Δ using a fluorescence polarization assay. Previously, we showed that the WT full-length Grp94 interacts weakly with Δ131Δ (40). Fluorescence polarization binding curves obtained with FITC-Δ131Δ K16C demonstrated that Grp94 H146D binds client with ∼6-fold higher apparent affinity than Grp94 WT (Fig. 5*D*). This supports the idea that client engagement by H146D hinders luciferase release by Grp94 for reactivation.

### The pre-N domain demonstrates client-specific effects

**Figure 7:**
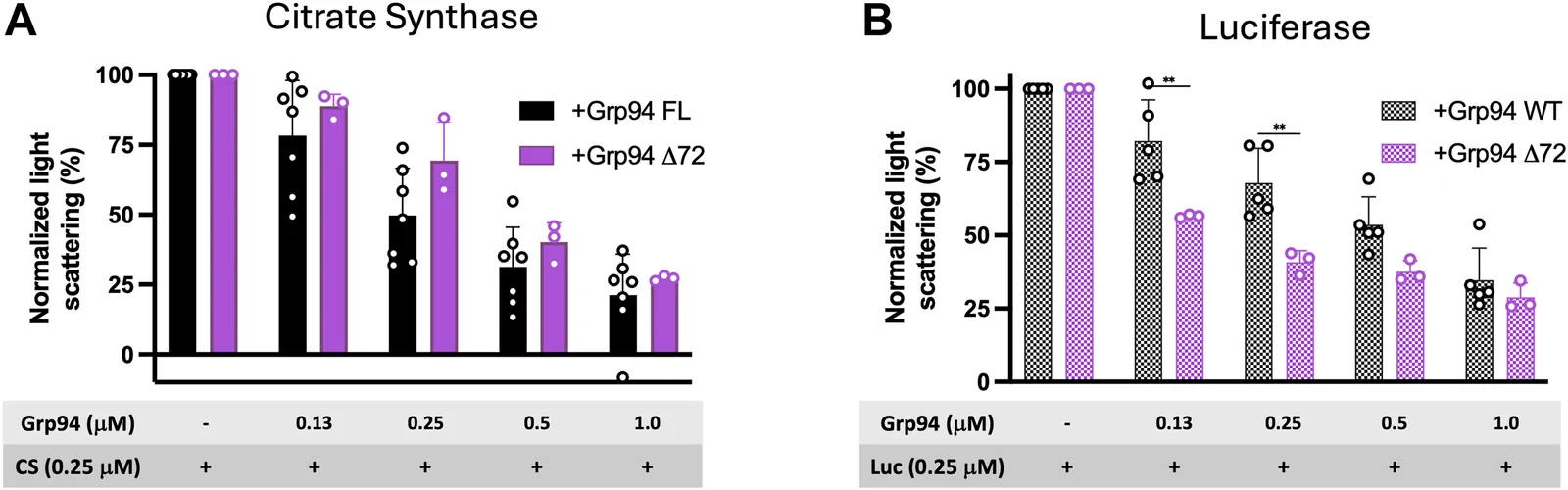
**The role ofGrp94’s pre-N domainin aggregation prevention is client specific.** Fixed-angle light scattering assays were carried out as described in Methods. *A*, CS or (*B*) luc (0.25 μM) was incubated at 45 °C for 30 min alone or in the presence of Grp94 WT and Grp94Δ72 (0.125 μM, 0.25 μM, 0.5 μM, 1 μM). The plateau values for each time trace were averaged and are shown in bar graphs as mean ± SD, with individual data points representing independent experiments. In (*A*), n = 7 for Grp94 WT and n = 3 for Grp94Δ72; in (*B*), n = 5 for Grp94 WT and n = 3 for Grp94Δ72. For (*A*) and (*B*), statistical variance was analyzed using ordinary two-way ANOVAs. For (A) the analysis revealed significant effects for the Grp94 concentration (F (4,40) = 48.12, *p* < 0.0001, Grp94 variant (F(1, 40) = 4.837, *p* = 0.0337) but no significant effect for the interaction between concentration and variant (F(4, 40) = 0.6142, *p* = 0.6549). Pairwise comparisons between Grp94 WT and Grp94Δ72 at each chaperone concentration were performed using Tukey’s multiple comparisons test; all comparisons within each concentration showed no statistically significant differences (*p* > 0.1766 across all pairs). For (*B*), the analysis revealed significant effects for the Grp94 concentration (F (4,30) = 66.61, *p* < 0.0001), Grp94 variant (F(1, 30) = 27.16, *p* < 0.0001) and interaction between concentration and variant (F(4, 30) = 3.466, *p* = 0.0192). Pairwise comparisons within each Grp94 concentration group were performed using Tukey’s multiple comparisons test; significant individual pairs are indicated by *asterisks*: ∗∗*p* < 0.01 (explicitly, *p* = 0.0019 and *p* = 0.001, respectively). All other comparisons within each concentration were non-significant (*p* > 0.0859). CS, citrate synthase; Luc, luciferase.

In previous studies from the Lieberman lab, a region in Grp94’s NTD was shown to promote aggregation of the client protein myocilin, which could be reverted by removing the pre-N domain or through Grp94 inhibition (33). Crosslinking studies identified residues of Grp94, including K72 of the pre-N domain and K140 of the NTD, that interact with the client (33). For model clients like luciferase and citrate synthase, we did not observe enhanced aggregation in the presence of Grp94. However, Grp94 H146, which is separated from K140 by ∼10 Å, functioned modestly better in the prevention of aggregation compared to the Grp94 WT. Given the proximity between Grp94 H146 and K140, we wondered whether a common surface of Grp94 interacts with client proteins and if the pre-N domain is involved in interactions. We tested the Grp94 construct lacking the pre-N domain (Grp94Δ72) for its ability to prevent aggregation. Grp94Δ72 appeared to be slightly less effective at preventing aggregation of CS, though the results were not statistically significant (Figs. 7*A* and S9, *A*, *B*). In contrast, the ability of Grp94Δ72 to prevent thermal aggregation of luciferase is enhanced at sub-stoichiometric or stoichiometric amounts of chaperone (Fig. 7*B*). Taken together, the pre-N domain did not play a significant role in preventing or promoting aggregation of these two model client proteins. Interactions between clients and Grp94’s pre-N domain and the resulting effects on aggregation are likely client specific.

## Discussion

Hsp90 is widely recognized as a classical “holdase”, functioning in stabilizing unfolded polypeptides and preventing protein aggregation (9, 48, 61, 62, 63). These activities have also been extended to Grp94, though the requirements are not fully understood. In this work, we show that the NTD of Grp94 is required to suppress client aggregation. Using model clients including citrate synthase and luciferase, full-length Grp94 and its isolated NTD efficiently prevent both heat and chemically induced aggregation in a concentration-dependent manner, thereby maintaining client solubility for later reactivation (9, 64). Notably, ATP binding and hydrolysis, calcium binding, and the conformational state do not have strong effects on Grp94’s prevention of aggregation activity, indicating that prevention of aggregation is largely nucleotide and calcium-independent, requiring neither a specific closed conformation of Grp94 nor complex ATP-driven structural changes. In contrast, our previous work showed that one mode of collaboration between Grp94 and BiP, termed the holdase-first pathway, occurred by the prevention of client aggregation by Grp94 followed by subsequent remodeling by BiP (46). Under these conditions, Grp94 required ATP binding but not necessarily ATP hydrolysis (46). This apparent discrepancy in Grp94’s requirements is reconciled by isolating the first step of the two-step chaperone mechanism (prevention of aggregation), omitting the subsequent client release step. In the absence of a downstream chaperone system, Grp94 prevents the formation of insoluble aggregates by binding to non-natively folded clients in an ATP-independent manner (9, 64). While productive refolding ultimately relies on client dissociation from Grp94, possibly initiated by ATP-binding (40), the isolated aggregation prevention step holds clients in a reactivatable, folding-competent state.

This study emphasizes the importance of a peptide-binding region in Grp94’s NTD in its prevention of aggregation activity. The peptide-binding mutant, H146D, is more effective at preventing aggregation at substoichiometric or equimolar chaperone-to-client concentrations. At these low chaperone-to-client ratios, chaperone efficiency is paramount to suppress aggregation. In contrast, at higher ratios, the total binding capacity of the chaperone pool is no longer a limiting factor, diminishing this functional advantage. Consequently, in the holdase-first pathway, the presence of Grp94 H146D results in decreased client remodeling.

Conversely, we previously characterized a second mode of collaboration for BiP and Grp94, the active-foldase pathway that proceeds in the reverse direction and begins with remodeling by BiP followed by ATP-dependent remodeling by Grp94. In this pathway, the peptide-binding mutant not only abolishes the 10% increase in remodeling activity observed when Grp94 WT was added but also stalls productive folding by BiP. When evaluating binding affinity, Grp94 H146D demonstrates tighter binding compared to Grp94 WT. Intriguingly, the behavior of H146D mirrors that of the Grp94 ATP-binding mutant, D149N, which similarly stalls productive folding and results in a stronger client affinity (40). In this active-folding context, the client binding region is localized to the dimer interface involving the CTDs and MDs of Grp94 (28, 29, 30, 65, 66). Structurally, the ATP-lid is open in the nucleotide-free state and is hypothesized to localize near the client-binding region to form a continuous surface (40). ATP binding by Grp94 induces a conformational change in the ATP-lid, resulting in the lid closing over the ATP binding pocket and moving away from the client binding surface, thus reducing client binding affinity (40). Because the D149N mutant cannot undergo this structural transition, it remains trapped in the high affinity conformation where refolding cannot occur (67). Thus, while the behavior of Grp94’s ATP-binding mutant likely involves structural effects, the driving factor behind the behavior of Grp94 H146D remains unclear. Nonetheless, the results from functional assays suggest that both mutations alter client affinity by decreasing client dissociation rates, effectively preventing client release from Grp94 required for BiP to complete effective remodeling.

The increased affinity of the Grp94 H146D mutant raises the question of whether this site defines the location of direct client interaction or whether its mutation results in an allosteric effect. Intriguingly, our previous work showed that residues around the ATP-binding pocket, including H146, are involved in allosteric networking of global conformational changes (68). Yet, a striking divergence between the behavior of the peptide binding mutant and the ATP-binding mutant emerges in functional assays. In the active-foldase pathway, both mutants were defective in remodeling and inhibited remodeling by BiP, resulting in an identical high-affinity client bound state. In contrast, in the holdase-first pathway, the mutants behave differently, suggesting divergent mechanisms. Grp94 D149N behaved similarly to Grp94 WT in ATP-independent prevention of aggregation assays, while Grp94 H146D demonstrated enhanced activity, suggesting that this may be a site of direct interaction. Additional support for a direct interaction is derived from crosslinking studies that have indicated regions on the pre-N domain and NTD of Grp94, including K140, interact with aggregation-prone clients (33). H146 and K140 are spatially situated in distinct clefts of the NTD, separated by ∼10 Å, and partially accessible in both the active and relaxed conformations. Furthermore, regions in the NTD of other Hsp90s have been similarly implicated in client binding (21, 62, 69). Collectively, these structural similarities and functional divergences support a mechanism in which the peptide-binding region of Grp94 is a site of direct client interaction during aggregation prevention. However, they do not rule out the possibility of complex allosteric effects or alternative mechanisms.

While Grp94’s NTD is sufficient for preventing aggregation, full-length Grp94 is required to support client refolding and activation in collaboration with BiP. Even in the prevention of aggregation assays, the isolated NTD exhibits a structural variance in the presence of the client. While the NTDs could maintain the client in a soluble state, the NTDs were less effective at preventing the accumulation of larger soluble intermediates. Two mechanistic possibilities may explain these behaviors. In the first scenario, the stoichiometry of one client to one Grp94 dimer is suggested, whereby the NTD is the primary site of client interaction but the MD and CTD transiently interact with the client protein to shield it from off-target interactions. This is supported by the observation that MD and CTDs are recruited to the client aggregates, yet the independent domains do not confer prevention of aggregation activity. Alternatively, when the client is exposed to NTDs, the 1:1 stoichiometry is lost and multiple NTDs can dock onto different regions of the client and hold it in an extended state. This could explain the larger soluble complexes observed by DLS in the presence of Grp94 NTDs. In this scenario, too many Grp94 NTDs may stall productive client folding, similar to the kinetic trapping observed with high concentrations of Hsp70’s (43). In both scenarios, communication and rearrangement between domains may be required to initiate client release for productive downstream remodeling. Taken together, these observations underscore that NTD-mediated aggregation suppression operates not in isolation but may likely work as part of a broader network of coordinated interdomain interactions within the full-length protein.

In contrast to Grp94’s protective activities, under specific conditions, Grp94 exhibits pro-aggregation activity (33). Grp94 was shown to stabilize the unfolded state of the client proIGF2, which under ADP conditions actually promoted proIGF2 oligomerization (70). In another study, Grp94 was shown to recognize on-pathway aggregates of mutant myocilin, a Grp94 client involved in glaucoma, where Grp94 accelerates aggregation and co-aggregates with the client (71). Further studies showed the combined MD and CTD fragment of Grp94 was capable of preventing aggregation of mutant myocilin, while the NTD enhanced aggregation (33). Likely, this pro-aggregation activity represents a pathological, mutation-dependent outcome that has not been observed to occur with Grp94 WT or the isolated Grp94 NTD toward model clients. This suggests that aggregation promotion by Grp94 is likely not a general attribute of the chaperone but instead arises when client intrinsic stability and aggregation pathways are altered to favor amyloid formation and the accumulation of aberrant, kinetically trapped complexes (33, 72).

Grp94's domain-specific architecture parallels observations from antigen-presentation cells studies, where the NTD has been identified as the primary peptide-binding module, with a site within the first 355 amino acids that interacts with antigen-presentation cell receptors and facilitates MHC class I loading (37, 39). The structural organization of the NTD features a concave β-sheet surface with a deep hydrophobic pocket (38), suited to selective molecular recognition of diverse ligands, while the pre-N domain regulates conformational dynamics that likely influence how long bound ligands are retained during maturation (30, 39). The client-specific effects observed across the NTD and pre-N domains underscore the sophistication of Grp94's recognition system and its ability to coordinate proteostatic, immunological, and stress-response functions. A clearer understanding of these mechanisms will not only enhance our knowledge of ER proteostasis and immune regulation but also provide a conceptual framework for the rational design of selective Grp94 modulators with therapeutic potential in cancer, neurodegenerative disorders, and autoimmune diseases (73, 74, 75).

## Experimental procedures

### Plasmid construction

All plasmids encoding Grp94 constructs were derived from pET expression vectors for inducible protein production in *E. coli*. The WT full-length Grp94 (*homo sapiens*, residues 22–803) consists of three domains, including the N-domain (NTD, residues 22–287), a MD, residues 327–594), and a C-domain (CTD, residues 595–803). The gene encoding the WT full-length Grp94 sequence (residues 22–803) preceded by an N-terminal hexahistidine (His_6_)-SUMO tag followed by TEV protease cleavage site was custom synthesized and inserted into the pET15b vector at NdeI and XhoI restriction sites (Genscript). This plasmid was used as a template for subsequent truncation and domain constructs and for mutagenesis to produce Grp94 H146D (Table 1).

**Table 1:** Summary of primers, templates, and cloning strategies for Grp94 deletion and point mutant constructs

Constructonstruct	Primers	Template DNA	Restriction sites	Plasmid
His_6_-SUMO-TEV-Grp94 FL (resid 22–803)	n/a purchased from Genewiz		5′ NdeI 3′ XhoI	pET15b
His_6_-SUMO-TEV-Grp94 N (resid 22–803; 288 STOP)	288 stop Fwd: GAGCAGCAAGACTGAATAAGTTGAGGAGCCCATGGAGG 288 stop RC: CCTCCATGGGCTCCTCAACTTATTCAGTCTTGCTGCTC	Grp94 FL	5′ NdeI 3′ XhoI	pET15b
His_6_-SUMO-TEV-Grp94 NM (resid 22-803; 595 STOP)	595 stop Fwd: CCAGAATGTTGCCAAGGAATAAGTGAAGTTCGATGAAAG 595 stop RC: CTTTCATCGAACTTCACTTATTCCTTGGCAACATTCTGG	Grp94 FL	5′ NdeI 3′ XhoI	pET15b
His_6_-SUMO-TEV-Grp94 M (resid 327–803; 595 STOP)	NdeI-6his Fwd: ATATATCATATGAAATCTTCTCACCATC TEV-aa337 RC: GCCATATTGGTTTGATATCATTGGAGCCGGAGG TEV-aa337 F: CCTCCGGCTCCAATGATATCAAACCAATATGGC aa803-STOP-XhoI RC: ATATATCTCGAGTTACAATTCATCTTTTTCAGC	Grp94 NM	5′ NdeI 3′ XhoI	pET28b
His_6_-SUMO-TEV-Grp94 C (resid 581–803)	NdeI-6his Fwd: ATATATCATATGAAATCTTCTCACCATC TEV-aa581 RC: CCTCTTCCCATCAAATTCGGGGGAGCCGGAGG TEV-aa581 F: CCTCCGGCTCCCCCGAATTTGATGGGAAGAGG aa803-STOP-BamHI RC: ATATATGGATCCTTACAATTCATCTTTTTCAGC	Grp94 FL	5′ NdeI 3′ BamHI	pET15b
His_6_-SUMO-TEV-Grp94Δ72	NdeI-6his Fwd: ATATATCATATGAAATCTTCTCACCATC TEV-aa73 RC: GGAAGGCAAACTTTTCCGAGGAGCCGGAGG TEV-aa73 Fwd: CCTCCGGCTCCTCGGAAAAGTTTGCCTTCC aa803-STOP-BamHI RC: ATATATGGATCCTTACAATTCATCTTTTTCAGC	Grp94 FL	5′ NdeI 3′ BamHI	pET15b
His_6_-SUMO-TEV-Grp94H146D	H146D Fwd: GAAGAACCTGCTGGATGTCACAGACACCGG H146D RC: CCGGTGTCTGTGACATCCAGCAGGTTCTTC	Grp94 FL	5′ Nde1 3′ Xho1	pET15b

For each construct, the template DNA, respective forward and reverse primers, and cloning strategy is shown.

Truncation mutants encompassing the N and NM domains were generated from the full-length WT construct by site-directed mutagenesis. A stop codon was inserted at position 288 or 595, respectively, using the QuikChange Lightning kit (Agilent) according to the manufacturer’s instructions. Primers for the reactions are listed in Table 1.

The M and C domain constructs were generated independently using PCR overlap extension and gene splicing as previously described by Heckman & Pease (76). Briefly, custom oligonucleotide primers (Table 1) were designed to amplify two overlapping gene segments from the full-length Grp94 plasmid template. The first gene product incorporates the NdeI restriction site followed by the His_6_-SUMO-TEV tag and the 5′ sequence of the domain of interest (M or C). The second gene product begins with the 5′ sequence of the domain of interest and terminates at the C-terminus of the Grp94 sequence followed by a restriction site (XhoI or BamHI; Table 1). These two overlapping gene products are combined in a subsequent PCR reaction using flanking primers (NdeI and XhoI or BamHI) to yield the complete gene product of the domain of interest. The gel purified gene products and destination plasmids (pET15b or pET28b) were digested with their paired restriction enzymes. Following enzymatic digestion, the linearized vectors and gene fragments were ligated using Instant Sticky-end Ligase Master Mix containing a T4 DNA ligase (NEB). All constructed plasmids were confirmed by Sanger sequencing (Genewiz).

### Protein expression, purification and mutagenesis

WT and mutant full-length human Grp94 (aa 23–803), Grp94 lacking the pre-N domain (aa 73–803), Grp94 domains (NTD aa 22–287, MD aa 337–594, and CTD aa 581–803) were overexpressed from *E. coli* BL21, as described previously (46). Briefly, cells were grown at 37 °C and induced with IPTG overnight at 18 °C. Cells were lysed using a French press (14,000 psi), and the supernatant from the cell lysate was separated by a His-trap column (Cytvia). The His_6_-SUMO tag was cleaved by overnight dialysis with TEV protease at a 1:100 ratio. Excess TEV, cleaved tag, and any uncleaved Grp94 were removed with a His-trap column (Cytvia). Lastly, size exclusion chromatography was run using a SuperDex 16/600 HiLoad column (Cytvia). All Grp94 mutations were constructed using site-directed mutagenesis with the QuikChange Lightning Kit (Agilent). Protein concentrations were determined spectrophotometrically. In all functional assays, full-length or pre-N truncated Grp94 concentrations are calculated based on their respective dimeric molecular weights, while all domain constructs are calculated based on their respective monomeric molecular weights.

Human BiP (aa 27–650), human DnaJB11 (aa 23–358), and human Grp170 (aa 33–999) were purified as described previously (46). BiP, DnaJB11, and Grp170 concentrations are calculated based on their respective monomeric molecular weights.

Δ131Δ K16C was overexpressed in BL21(DE3) cells and purified as described previously (40). Briefly, after lysis, Δ131Δ was recovered from the insoluble fraction, refolded on a SP Sepharose column (Cytvia), and further purified using size exclusion chromatography with a SuperDex16/600 HiLoad column (Cytvia).

### Client protein preparation

Citrate synthase was purchased from Sigma-Aldrich as an ammonium sulfate suspension. The ammonium sulfate was removed by centrifugation at 10K RCF for 15 min at 4 °C. The remaining citrate synthase pellet was resuspended in an equal volume of buffer. For chemical denaturation, denaturation buffer was used (6 M Gdn-HCl, 100 mM Tris pH 7.5, 5 mM DTT), while a TE buffer (50 mM Tris pH 7.5, 2 mM EDTA) was used for thermal denaturation. Residual ammonium sulfate was removed by buffer exchange using polyacrylamide desalting columns (Pierce). Aliquots of prepared citrate synthase were frozen at −80 °C (chemical) (61) or −20 °C (thermal) (48, 77).

Luciferase was purchased from Sigma-Aldrich as a lyophilized solid and stored at −20 °C. For thermal denaturation, luciferase was dissolved in TE buffer (50 mM Tris pH 7.5, 2 mM EDTA) to a final concentration of 1 mg/ml. Aliquots of prepared luciferase were frozen at −20 °C.

### Fixed angle light scattering

Fixed angle light scattering was performed using a PerkinElmer LS 50B Luminescence Spectrometer using a sub-micro quartz fluorometer cell (Starna). Chemically denatured citrate synthase was prepared as described above. Grp94 was supplemented with 1 mM, 1 mM Ca^2+^ and 50 μM ATP for thermal denaturation. For experiments with CS alone, prepared CS was diluted into refolding buffer (25 mM Hepes pH 7.4, 50 mM KCl and 0.1 mM EDTA) to a final client concentration of 0.25 μM. For experiments containing chaperone, CS was diluted into refolding buffer containing Grp94 at the indicated concentrations and 50 μM ATP and 250 μM MgCl_2_ prior to the addition of chemically denatured citrate synthase. Light scattering signal was monitored over 7 min, with excitation and emission wavelengths at 550 nm and a slit width of 5.5 nm. Three or more independent experiments were conducted, as indicated in each figure legend. Data were analyzed using GraphPad Prism. Raw data from light scattering were input and the average buffer baseline was substracted. For the bar graphs, the light scattering signal for chemical denaturation was calculated as the average signal over 5 min from time trace plateau between the 2 to 7-min timepoints. The resulting signals were then normalized by setting the CS alone signal to 100%, and all chaperone-containing traces are presented relative to this maximum aggregation control. To determine if aggregation prevention by Grp94 was significant, statistical analysis was performed using a two-way ANOVA. Statistical significance is quantified as: ∗∗∗∗*p* < 0.0001; ∗∗∗0.0001 ≤ *p* < 0.001; ∗∗0.001 ≤ *p* < 0.01; ns, *p* ≥ 0.05.

For thermal denaturation, a circulating water bath was attached to the fluorometer, and the temperature of the cell holder was set to 45 °C. Prepared citrate synthase or luciferase was added to refolding buffer, with or without chaperones, to a final client concentration of 0.25 μM. Light scattering was monitored over 30 min, with excitation and emission wavelengths at 360 nm and a slit width of 2.5 nm. All experiments were performed in triplicate. Data were analyzed using GraphPad Prism. Raw data from light scattering were input and subsequently normalized by substracting the average buffer baseline. Average light scattering signal for heat denaturation was calculated as the average of the time trace plateau from 1500 to 1800 s and plotted using a bar graph for at least three experiments ±SD. When comparing different datasets, data from each experiment were normalized against the maximal signal of CS aggregation in the absence of chaperone. To determine if aggregation prevention by Grp94 was significant, statistical analysis was performed using a two-way ANOVA. Statistical significance is quantified as: ∗∗∗∗*p* < 0.0001; ∗∗∗0.0001 ≤ *p* < 0.001; ∗∗0.001 ≤ *p* < 0.01; ns, *p* ≥ 0.05.

### DLS

Dynamic light scattering measurements were performed on Zetasizer Nano S (Malvern Instruments). Chemically denatured CS, heat denatured CS, heat denatured luciferase, or native or denatured Grp94 individually or with CS/luciferase were equilibrated to 25 °C in measurement buffer (25 mM Hepes-NaOH pH 7.4, 50 mM KCl and 0.1 mM EDTA) prior to measurement. CS and luciferase were added at a concentration of 0.25 μM, and Grp94 was added at a concentration of 1 μM for all DLS experiments. At least three independent experiments were carried out per sample, as indicated in each figure legend.

### Citrate synthase activity assay

The activity of citrate synthase (CS) was monitored using a colorimetric assay based as previously described (48), with minor modifications. CS catalyzes the condensation of oxaloacetic acid (OAA) and acetyl-CoA to form citrate and coenzyme A (CoA-SH). The resulting CoA-SH reacts stoichiometrically with Ellman’s reagent (DTNB) to generate 5-thio-2-nitrobenzoic acid (TNB), which produces a measurable increase in absorbance at 412 nm. This change in absorbance is directly proportional to CS activity.

The CS was prepared as described above with additional purification using a Superdex 75 Increase 10/300 column (Cytvia) (78). A 15 μM stock solution of CS was then prepared and subsequently diluted 100-fold into 40 mM Hepes-KOH buffer pH 7.5, preincubated at 45 °C, in the presence or absence of Grp94 at a final concentration of 0.6 μM to initiate thermal inactivation. The reaction mixture was maintained at 45 °C throughout the inactivation period. After 20 min of inactivation, oxaloacetic acid (OAA; stock solution of 100 mM) was added at a 100-fold dilution to allow for the reactivation of folding intermediates. CS activity was monitored at t = 0, 5, 10, 20, 30, 40, and 60 min by transferring 4 μl of the inactivation reaction into 196 μl of preincubated (25 °C) reaction buffer (50 mM Tris pH 8.0, 2 mM EDTA, 0.1 mM OAA, 0.1 mM DTNB, and 0.15 mM acetyl-CoA). Enzymatic activity was measured by monitoring the increase in absorbance at 412 nm over 1 min using a BioTek Cytation five plate reader.

### Luciferase reactivation assay

Luciferase reactivation assays for Grp94 WT, domains and mutants were performed as described previously (40, 46). Briefly, luciferase (Promega) was denatured in the presence of chaperone(s) and ATP. These reactions were diluted into renaturation buffer (25 mM Hepes-KOH 7.5, 50 mM KCl, 0.1 mM EDTA, 10 mM MgCl_2_, 0.1 mM CaCl_2_, 50 ug/ml bovine serum albumin) containing an ATP-regenerating system (2 mM ATP, 20 mM phosphocreatine, and 60 μg/ml creatine kinase). Luciferase activity was measured by transferring 5 μl aliquots of the reaction mixture to a 96 well plate at the indicated time points and adding 120 μl of luciferin solution (25 mM Tris-HCl pH 7.8, 100 mM KCl, 10 mM MgCl_2_, 0.5 mM ATP, and 50 μg/ml luciferin). Luminescence was measured using a BioTek Cytation five plate reader.

For the prevention of aggregation by Grp94, 160 nM luc was heat denatured at 45 °C for 7 min in the absence or presence of 5 μM WT or mutant Grp94, cooled at 4 °C for 5 min, and diluted 10-fold into renaturation buffer containing the BiP chaperone system (2 μM BiP, 4 μM DnaJB11, and 0.2 μM Grp170).

In the collaborative refolding assay, 25 nM luc was heat denatured at 42 °C for 10 min in the presence of 2 μM BiP, 4 μM DnaJB11, 0.05 μM Grp170, and 1 μM Grp94. After heating, this reaction was diluted to a final luc concentration of 20 nM into renaturation buffer and incubated at 30 °C.

### CD

CD spectrometry was performed on an AVIV Model 435 CD Spectrometer. Samples of Grp94 were analyzed in a 1 mm path length quartz cuvette at 25 °C, and spectra were collected from 260-200 nm. Data points were collected every 1 nm with a 3 s averaging time. Using Amicon Ultracel 10k MWCO 0.5 ml concentrators, WT and mutant Grp94 were buffer exchanged into 10 mM Tris-HCl, 100 mM KCl, 5% glycerol and 0.2 mM TCEP. Grp94 was then diluted to 2 uM in 350 uL reaction buffer and samples were run in triplicate.

### Trypsin digestion

Trypsin digestion was performed as described previously (46). WT or mutant Grp94 (20 μg) was digested with trypsin (20 ng) at 37 °C in reaction buffer (25 mM Hepes-KOH, 50 mM KCl, and 2 mM DTT). 10 μl aliquots were removed at times 0, 5, 10, and 30 min, boiled in NuPAGE LDS buffer (Thermo Fisher Scientific) and run on a 4 to 20% Bis-Tris gel (Genscript). Gels were visualized by Coomassie blue staining overnight at room temperature, destained in deionized water and imaged on a Bio-Rad ChemiDoc MP imaging system.

### Native PAGE gel electrophoresis

The native conformation and oligomeric state of the purified NTD, MD, and CTD of Grp94 were analyzed by native PAGE using the NativePAGE Novex Bis-Tris system (Invitrogen). For each domain, 5 μg of protein was mixed with 1 × native sample buffer without detergent or additives, kept on ice, and resolved on a 3 to 12% Bis-Tris gel alongside an unstained native molecular weight marker. Electrophoresis was carried out in an XCell SureLock Mini-Cell 1 × running buffer at 150 V for ∼90 to 115 min with pre-chilled buffers, and proteins were visualized by Coomassie blue staining overnight at room temperature. Gels were then destained in deionized water and imaged on a Bio-Rad ChemiDoc MP imaging system.

### Fluorescence polarization

Assays were carried out as described previously. Δ131Δ was labeled with 3-fold excess FITC (GoldBio) at room temperature for 45 min. Excess label was removed using 0.5 ml Zeba desalting columns (Thermo Fisher Scientific). Grp94 was concentrated and buffer exchanged into assay buffer containing 25 mM Hepes-KOH pH 7.4, 100 mM KCl, 0.01% and Triton X-100. 80 μM of Grp94 WT or H146D was serially diluted and incubated with Δ131Δ K16C (50 nM) on a 384-well, black, low-volume, round-bottom plate (Corning) for 30 min at 37 °C in the dark. Steady-state polarization was measured using a BioTek 5 Cytation plate reader (Ex: 485/20 nm; Em: 528/20 nm). Data were analyzed using Graphpad Prism 10 (https://www.graphpad.com/) and fitted using a one-site specific binding model.

## Data availability

All data generated by this study are available within this article and the supporting information.

## Supporting information

This article contains supporting information.

## Conflicts of interest

The authors declare that they have no conflicts of interest with the contents of this article.
