# Supplementary material for: The N-terminal domain of the endoplasmic reticulum-resident chaperone Grp94 functions in aggregation prevention
Source: J Biol Chem. 2026 Jul 22;302(9):113358. doi: 10.1016/j.jbc.2026.113358 (PMC13507752; doi:10.1016/j.jbc.2026.113358)
Supplement: Supplementary file 1 [file mmc1.docx]

**Supplementary Information:**

**The N-terminal domain of the endoplasmic reticulum-resident chaperone Grp94 functions in aggregation prevention**

**Authors & Affiliations**

Ayodeji Adedeji^a^, Erin Unruh^a,b^, Alyssa Derr^a^, Chioma Ndolo^a^, Melina Balch^a^, Kylie Paul^a,c^, AJ Morton^a,d^, and Andrea N. Kravats^a,b,e^

a. Department of Chemistry & Biochemistry, Miami University, Oxford, OH 45056

b. Cell, Molecular, and Structural Biology Graduate Program, Miami University, Oxford, OH 45056

c. Current Affiliation: Molecular and Cellular Biology Graduate Program, University of Massachusetts Amherst, Amherst, MA 01003

d. Current Affiliation: Biochemistry Graduate Program, Indiana University, Bloomington, IN 47401

e. Corresponding author: kravatan@miamioh.edu

**Figure S1**: **Effect of Grp94’s prevention of aggregation of citrate synthase on citrate synthase activity.** Citrate Synthase (CS) (1.5 nM) was heat denatured in the absence or presence of 6 nM Grp94. Reaction aliquots were removed from heat and CS activity was monitored through the reaction catalyzed in the citric acid cycle. Native CS activity is set to 100%. For the other reactions, the average activity (slopes) measured over a one-minute interval at the indicated time points are displayed as a percentage of CS activity. Data are presented as mean ± SD (n = 3 independent experiments).

**Figure S2: Citrate synthase aggregation is prevented by Grp94 in the presence of calcium, EGTA, and ATP**.

(A, C, E, G) Citrate synthase (CS) (0.25 µM) was incubated at 45 °C for 30 minutes alone or in the presence of Grp94 (0.125µM, 0.25µM, 0.5µM, or 1 µM), and 90° light scattering was recorded as a function of time. A representative trace from three experiments is shown for Grp94 in the presence of calcium (A), EGTA (C), ATP (E), or ATP and calcium (G). (B, D, F, H) Dynamic light scattering of thermally denatured CS (0.25 µM) with Grp94 (1 µM) in the presence of calcium (B), EGTA (D), ATP (F), or ATP and calcium (H). Data are presented as mean of independent experiments (n = 19 for native or heated CS; n = 3 for all other conditions).

**Figure S3: Characterization of Grp94 mutants**

(A) Crystal structure of Grp94 in the relaxed conformation (PDB ID 2O1V) with one protomer in grey and the other protomer in color (N-terminal domain (NTD) in cyan, middle domain (MD), in green, and C-terminal domain (CTD) in dark blue) using a surface model. Residues H146 (dark red) and K140 (orange) are surface exposed, while D149 (dark green) and E103 (magenta) are involved in coordinating nucleotide and nucleotide hydrolysis (inset). The pre-N domain was not resolved in this structure. (B) Circular dichroism (CD) spectra of Grp94 wild-type and mutant as described in Methods. Spectra are represented as the mean ± SD of six independent experiments for Grp94 WT and D149N, and three for E103A and H146D. (C) Trypsin digestion of Grp94 WT or mutant (20 µg) as described in Methods. At the indicated times, aliquots were removed and analyzed by SDS-PAGE. Data are from a single experiment.

**Figure S4: Citrate synthase aggregation is prevented by Grp94 defective in ATP binding, Grp94 defective in ATP hydrolysis or Grp94 trapped in the catalytically active conformation.**

(A, C, E) Citrate Synthase (CS) (0.25 μM) was incubated at 45^o^C for 30 minutes alone or in the presence of Grp94 (0.125 μM, 0.25 μM, 0.5 μM, and 1 μM) and 90^o^ light scattering was recorded as a function of time. A representative trace from three experiments is shown for Grp94 E103A (A), Grp94 D149N (C), or Grp94 preincubated with AMP-PNP (E). (B, D, F) Dynamic light scattering of thermally denatured CS (0.25 μM) with 1 μM Grp94 E103A (B), Grp94 D149N (D) or Grp94 preincubated with AMP-PNP (F). Data are presented as mean of independent experiments (n = 19 for native or heated CS; n = 3 for all other conditions).

**Figure S5: Oligomerization of Grp94 domains**

Purified N-terminal domain (NTD), middle domain (MD), and C-terminal domain (CTD) of Grp94 were resolved under non-denaturing conditions on a NativePAGE Novex® Bis-Tris gel (3-12%). Each lane was loaded with 5 µg of protein. A molecular weight marker (MWM) is shown in the leftmost, lane, with molecular weights indicated (kDa). The gel shown is representative of two independent experiments.

**Figure S6: The N-terminal domain of Grp94 is responsible for citrate synthase aggregation prevention.**

(A-C) Citrate Synthase (CS) (0.25 μM) was incubated at 45^o^C for 30 minutes alone or in the presence of Grp94 (0.125 μM, 0.25 μM, 0.5 μM, and 1 μM) and 90^o^ light scattering was recorded as a function of time. Representative traces from three experiments are shown for Grp94 NTD (A), Grp94 MD (B), and Grp94 CTD (C).

**Figure S7: The N-terminal domain of Grp94 is responsible for luciferase aggregation prevention.**

(A–D) Luciferase (Luc) (0.25 μM) was incubated at 45^o^C for 30 minutes alone or in the presence of Grp94 (0.125 μM, 0.25 μM, 0.5 μM, and 1 μM) and 90^o^ light scattering was recorded as a function of time. Representative traces from one of three experiments are shown for full-length Grp94 (A), Grp94 NTD (B), Grp94 MD (C), or Grp94 CTD (D).

**Figure S8: The peptide binding mutant of Grp94 exhibits enhanced aggregation prevention at limiting chaperone concentrations.**

Fixed-angle light scattering assays were carried out as described in Methods. Citrate synthase (CS) (A) or luciferase (luc) (B) (0.25 μM) was incubated at 45^o^C for 30 minutes in the absence or presence of wild type (WT) Grp94 or Grp94 H146D (0.125 μM, 0.25 μM, 0.5 μM, or 1 μM) and 90^o^ light scattering was recorded as a function of time. Representative traces from five experiments are shown for the prevention of aggregation of citrate synthase (A) or luciferase (C). (B, D) Dynamic light scattering (DLS) of Grp94 (1 µM) with heat-denatured citrate synthase (0.25 µM) (B) or luciferase (0.25 µM) (D). Data are presented as mean of independent experiments: for (B), n = 19 for native or heated CS and n = 3 for all other conditions; for (D), n = 9 for native or heated luciferase and n = 6 for Grp94 H146D.

**Figure S9: The role of Grp94’s pre-N domain in aggregation prevention is client specific.**

Fixed-angle light scattering assays were carried out as described in Methods. Citrate Synthase (CS) (0.25 μM) was incubated at 45^o^C for 30 minutes in the absence or presence of wild type (WT) Grp94 or Grp94Δ72 (0.125 μM, 0.25 μM, 0.5 μM, or 1 μM) and 90^o^ light scattering was recorded as a function of time. Representative traces from three independent experiments are shown for the prevention of aggregation of CS (A) or luciferase (C). (B, D) Dynamic light scattering (DLS) of Grp94 (1 µM) with heat-denatured citrate synthase (0.25 µM) (B) or luciferase (0.25 µM) (D). Data are presented as mean of independent experiments: for (B), n = 19 for native or heated CS and n = 3 for all other conditions; for (D), n = 9 for native or heated luciferase and n = 6 for Grp94Δ72.
